# An Uncertainty-Aware Temporal Transformer for Probabilistic Interval Modeling in Wind Power Forecasting

**DOI:** 10.3390/s26072072

**Published:** 2026-03-26

**Authors:** Shengshun Sun, Meitong Chen, Mafangzhou Mo, Xu Yan, Ziyu Xiong, Yang Hu, Yan Zhan

**Affiliations:** 1 School of Automation, Qingdao University, Qingdao 266071, China; 2 National School of Development, Peking University, Beijing 100871, China; 3 China Agricultural University, Beijing 100083, China; 4 Artificial Intelligence Research Institute, Tsinghua University, Beijing 100084, China

**Keywords:** AI-driven data analytics, data mining for energy systems, deep learning for power systems, wind power forecasting, temporal transformer

## Abstract

Under high renewable energy penetration, wind power forecasting faces pronounced challenges due to strong randomness and uncertainty, making conventional point-forecast-centric paradigms insufficient for risk-aware and reliable power system scheduling. An uncertainty-aware temporal transformer framework for wind power forecasting is presented, integrating probabilistic modeling with deep temporal representation learning to jointly optimize prediction accuracy and uncertainty characterization. Crucially, rather than treating uncertainty quantification merely as a post-processing step, the central conceptual contribution lies in modularizing uncertainty directly within the attention mechanism. A probability-driven temporal attention mechanism is incorporated at the encoding stage to emphasize high-variability and high-risk time slices during feature aggregation, while a multi-quantile output and interval modeling strategy is adopted at the prediction stage to directly learn the conditional distribution of wind power, enabling simultaneous point and interval forecasts with statistical confidence. Extensive experiments on multiple public wind power datasets demonstrate that the proposed method consistently outperforms traditional statistical models, deep temporal models, and deterministic transformers, as validated by formal statistical significance testing. Specifically, the method achieves an MAE of 0.089, an RMSE of 0.132, and a MAPE of 10.84% on the test set, corresponding to reductions of approximately 8%–10% relative to the deterministic transformer. In uncertainty evaluation, a PICP of 0.91 is attained while compressing the MPIW to 0.221 and reducing the CWC to 0.241, indicating a favorable balance between coverage reliability and interval compactness. Compared with mainstream probabilistic forecasting methods, the model further reduces RMSE while maintaining coverage levels close to the 90% target, effectively mitigating excessive interval conservatism. Moreover, by adaptively generating heteroscedastic intervals that widen during high-volatility events and narrow under stable conditions, the model achieves a highly focused and effective capture of critical uncertainty information.

## 1. Introduction

Under the dual-carbon goals and the development of new-generation power systems, the share of renewable energy in the electricity mix has continued to grow, among which wind power has become a major contributor due to its wide resource distribution, technological maturity, and strong scalability [1]. However, wind power output is highly dependent on wind speed, which is jointly affected by meteorological systems, terrain characteristics, and seasonal variations, leading to pronounced randomness, non-stationarity, and time-varying uncertainty [2]. These characteristics induce severe power fluctuations and pose substantial challenges to power system dispatch, secure operation, and reserve capacity planning [3]. As a key enabling technology for renewable energy integration, wind power forecasting plays a critical role in maintaining system stability and improving renewable energy utilization [4]. Consequently, achieving accurate and reliable wind power forecasting under complex and uncertain conditions remains a core research problem in power systems and energy artificial intelligence [5]. Early wind power forecasting studies primarily relied on physical and statistical models [6]. Physics-based approaches model the wind-to-power conversion process using turbine aerodynamics, power curves, and atmospheric dynamics, combined with numerical weather prediction data to jointly model wind fields and turbine operating states [7,8,9]. These approaches offer strong theoretical interpretability and are valuable for long-term planning [10]. However, their applicability is often constrained by the need for accurate turbine parameters and high-quality meteorological inputs, which amplifies forecasting errors in short-term scenarios and restricts their use in real-time dispatch [11,12]. Statistical methods, including time series and regression models, exploit historical power patterns and have shown effectiveness in short-term forecasting, offering computational efficiency in data-scarce settings [13,14,15,16]. However, most of these models rely on linear assumptions or weak nonlinear representations, limiting their ability to capture abrupt variations and complex nonlinear dynamics, leading to rapid performance degradation under extreme weather conditions [17,18]. More importantly, these methods are typically designed for point forecasting and fail to characterize predictive uncertainty, which restricts their usefulness in risk-aware system operation and probabilistic dispatch [19,20].

With increasing computational capability and data availability, machine learning and deep learning techniques have become dominant in wind power forecasting research [21,22]. Traditional machine learning models, such as support vector machines and random forests, improve nonlinear modeling capacity but remain highly dependent on handcrafted features and offer limited temporal dependency modeling [23,24,25]. Deep learning methods have subsequently introduced a new paradigm by enabling automatic learning of high-dimensional, nonlinear, and multi-scale representations [26,27]. Convolutional neural networks (CNNs) effectively extract local temporal patterns and spatial correlations from multivariate time series [28,29]. Recurrent neural networks (RNNs) and their variants, such as long short-term memory (LSTM) and gated recurrent unit (GRU) architectures, explicitly model temporal evolution and mitigate long-term dependency issues via gated mechanisms, achieving promising short-term forecasting performance [30,31,32,33]. Nevertheless, recurrent structures suffer from sequential computation and limited parallelism, and their memory mechanisms often fail to retain critical information under highly non-stationary conditions, making predictions sensitive to noise [34,35]. Transformer-based temporal models have recently gained attention due to the ability of self-attention mechanisms to capture long-term dependencies without sequential bottlenecks [36,37,38]. By modeling global interactions among arbitrary time steps, transformers exhibit strong capability in multi-scale temporal representation and dynamic pattern learning [39,40]. However, most existing transformer-based wind power forecasting methods adopt deterministic modeling paradigms, using transformers mainly as feature extractors or regressors that produce only point predictions [41,42,43]. Under highly volatile conditions, such deterministic outputs fail to reflect actual operational risks. In addition, conventional attention mechanisms typically treat all time steps uniformly, without explicitly accounting for variations in fluctuation intensity, resulting in insufficient sensitivity to high-uncertainty periods [44,45].

To overcome these limitations, probabilistic forecasting and uncertainty modeling methods have attracted growing interest to characterize the distributional properties of predictions rather than relying solely on point estimates [46,47,48]. Techniques such as quantile regression [49], interval prediction [50], and probabilistic distribution modeling [51] provide partial descriptions of forecasting uncertainty and support risk-aware scheduling [52]. While these approaches offer richer information for risk assessment, their effectiveness strongly depends on the validity of distributional assumptions, which are difficult to satisfy in complex wind power scenarios [53]. Moreover, integrating probabilistic forecasting with deep temporal architectures such as transformers remains challenging, preventing the establishment of a unified framework that jointly optimizes accuracy and uncertainty representation [54,55]. To systematically synthesize the current research landscape, Table 1 provides a quantitative and architectural comparison of representative wind power forecasting modeling paradigms, highlighting their specific metrics, reported performance scales, and primary technical limitations.

Recent studies have explored uncertainty-aware transformers in energy systems, including the knowledge transformer with uncertainty (KTU) for peer-to-peer energy trading [56], the GridFusionX framework for probabilistic load forecasting [57], spatiotemporal forecasting under stability constraints [58], and generative scenario forecasting via ScenGAN [59]. Despite these advances, a critical scientific gap remains in wind power forecasting: existing deep probabilistic models typically treat uncertainty quantification as a detached post-processing step or a separate decoding head, while the core temporal representation learning (i.e., the self-attention mechanism) remains fundamentally deterministic and oblivious to local volatility. Consequently, these models uniformly aggregate temporal features regardless of varying risk levels, failing to adaptively prioritize highly non-stationary and chaotic meteorological regimes. To bridge this gap, an uncertainty-aware temporal transformer-based wind power forecasting method is proposed, which fundamentally redesigns the attention mechanism to internalize heteroscedastic uncertainty directly into the sequence encoding process. The main contributions of this study are summarized as follows:

1. A novel uncertainty-aware temporal transformer framework is proposed, structurally resolving the disconnect between deterministic feature extraction and probabilistic forecasting by embedding risk perception directly into the deep representation learning pipeline;
2. A probability-driven temporal attention mechanism is formulated, which dynamically modulates the standard semantic inner-product similarity using a local root mean square fluctuation intensity, enabling the model to explicitly prioritize high-volatility, high-risk temporal slices during information aggregation;
3. A structurally constrained quantile prediction and interval modeling module is constructed, integrating time-slice and channel-correlation graph convolutional networks with a monotonic parameterization to capture complex multi-step variable coupling while mathematically preventing quantile crossing;
4. Extensive experiments on multiple public wind power datasets demonstrate that the proposed method overcomes the traditional trade-off in probabilistic forecasting, successfully generating heteroscedastic intervals that adaptively widen during extreme fluctuation scenarios while maintaining exceptional compactness and high coverage reliability.

## 2. Materials and Method

### 2.1. Data Collection

Multiple publicly available benchmark datasets for wind power forecasting are employed to conduct experimental studies, in order to ensure the reproducibility of experimental results and the fairness of comparisons among different methods, as shown in Table 2. First, the wind integration dataset released by the National Renewable Energy Laboratory (NREL) of the United States is adopted. Specifically, the NREL Wind Integration National Dataset (WIND) Toolkit is utilized, from which data for 10 geographically distributed wind farms within the Texas region are extracted to ensure diversity in terrain and microclimate. The selected subset covers a precise temporal span of three continuous years, from 1 January 2010 to 31 December 2012. This dataset is constructed based on real wind farm operational records and numerical meteorological simulation results, covering multiple wind farm sites and long temporal spans, and is therefore capable of effectively reflecting the temporal fluctuation characteristics of real-world wind power output. The data are typically sampled at fixed temporal resolutions, with sampling intervals of 5 min, and include multi-year continuous records of wind power generation together with corresponding meteorological driving variables. Wind power values are provided in a normalized form with respect to the rated capacity of each wind farm, which facilitates unified modeling and comparative analysis across different sites. The meteorological variables mainly include wind speed and wind direction at different heights, air temperature, and atmospheric pressure. These variables are obtained through numerical weather prediction models or on-site measurements and are strictly aligned with the wind power time series along the temporal dimension.

In addition, the wind power forecasting dataset released in the GEFCom2014 Global Energy Forecasting Competition is also utilized. In this study, the specific data from Task 15 of the Wind Forecasting track is employed, focusing exactly on Zones 1, 2, and 3. The extracted temporal coverage strictly spans two continuous years, from 1 January 2012 to 31 December 2013. This dataset is recognized as one of the classical benchmarks in the field of probabilistic wind power forecasting and has been widely used for evaluating the performance of uncertainty modeling methods. The GEFCom2014 wind dataset focuses on a single or a small number of wind farms and provides high-resolution historical power generation time series together with corresponding meteorological forecast information. The temporal coverage spans multiple consecutive months or years, enabling the representation of non-stationary and stochastic wind power characteristics under different seasonal conditions. Similar to the NREL dataset, wind power and meteorological variables in the GEFCom2014 dataset are provided in structured numerical form, without any image-based or spatial gridded information, which is more consistent with practical power system operation and dispatch scenarios that rely on SCADA measurements and numerical meteorological data.

To strictly prevent temporal data leakage during model training and evaluation, a chronological data partitioning policy is enforced across all datasets. The time series for each wind farm is partitioned into training, validation, and testing sets following a strict 70%:10%:20% chronological sequence. Random shuffling across the temporal dimension is strictly prohibited before the split to ensure that the model is evaluated solely on unseen future data, thereby accurately reflecting real-world sequential forecasting conditions.

### 2.2. Data Preprocessing and Augmentation Strategy

Wind power time series inevitably suffer from sensor failures, communication delays, and extreme operating conditions during real-world data acquisition, which results in missing values, outliers, and scale inconsistencies in the raw data. If such data are directly used for model training without proper processing, noise interference is likely to be introduced, leading to biased parameter estimation and consequently degrading forecasting performance and the stability of uncertainty modeling. Therefore, before constructing wind power forecasting models, systematic data preprocessing and data augmentation are required to enhance model robustness under complex operating conditions and stochastic fluctuations. In wind power time series, missing data usually appear as empty or invalid records of power output or meteorological variables at certain time steps. Improper handling of missing values may destroy the temporal continuity of the series and adversely affect the ability of temporal models to learn dynamic evolution patterns. Considering that wind power typically exhibits strong temporal correlation at short time scales, a time-neighborhood-based interpolation strategy is adopted for missing value imputation. Let the original time series be denoted as ${x_{t}}_{t=1}^{T}$. When the observation at time step *t* is missing, the value can be estimated using its neighboring valid observations, which can be expressed as

(1) $${\hat{x}}_{t}=\frac{1}{|N_{t}|}\sum_{k\in N_{t}}x_{k}$$

where $N_{t}$ denotes the set of valid neighboring indices around time step *t*, and $|N_{t}|$ represents the number of valid samples within the neighborhood. Specifically, a symmetric neighborhood with a maximum size of $\left|N_{t}\right|=4$ (i.e., the two closest valid observations before and after *t*) is utilized. For boundary cases at the beginning or end of the series, the neighborhood is simply truncated to include only the available valid side. This method preserves the smoothness of the time series without introducing additional assumptions and reduces the disruption of missing data to the overall temporal structure. In addition to missing values, outliers are another common issue in wind power data. Outliers may arise from sensor errors, communication abnormalities, or temporary turbine malfunctions, and their values often deviate significantly from normal operating ranges. If directly used for model training, outliers may be mistakenly learned as normal patterns, thereby impairing forecasting stability. To address this issue, outliers are identified and removed based on robust statistical distribution characteristics. Assuming that the power time series follows an approximately stable distribution within a local time window of length W=144 (corresponding to 24 h of 10-min resolution data), the robust median (Med) and Median Absolute Deviation (MAD) can be calculated, and observations exceeding a reasonable range are regarded as outliers. The detection criterion can be expressed as

(2) $$|x_{t}-\mathrm{Med}|>\alpha \mathrm{MAD}$$

where α is a threshold coefficient controlling the sensitivity of outlier detection, which is empirically set to α=3 in this study to reliably filter extreme anomalies without discarding natural volatility peaks. Once an observation is identified as an outlier, it is replaced using the same neighborhood interpolation strategy as that adopted for missing values, thereby removing abnormal noise while preserving temporal continuity. After missing value imputation and outlier processing, discrepancies in variable scales may still adversely affect model training. For example, wind speed, wind direction, and power output differ significantly in numerical ranges, and without appropriate treatment, gradient imbalance may occur during optimization. To improve training stability and convergence speed, normalization is applied to all input variables. Let the original variable be denoted as $x_{t}$, and its normalized form ${\tilde{x}}_{t}$ is given by

(3) $${\tilde{x}}_{t}=\frac{x_{t}-x_{\min}}{x_{\max}-x_{\min}}$$

where $x_{\min}$ and $x_{\max}$ represent the minimum and maximum values of the corresponding variable computed strictly on the training dataset. This parameter isolation is strictly enforced to ensure that no future information from the validation or testing sets leaks into the preprocessing phase. Through normalization, all variables are mapped to a unified scale, which facilitates stable learning of relationships among different features under multivariate input conditions. After data preprocessing, continuous time series need to be transformed into sample representations suitable for supervised learning. Wind power forecasting is essentially a problem of inferring future states from historical observations, and a sliding window mechanism is adopted to construct temporal samples. Specifically, let the historical window length be *L* and the forecasting horizon be *H*. At time step *t*, the model input consists of multivariate observations from the past *L* time steps, and the prediction target corresponds to the wind power sequence over the next *H* time steps. This process can be formulated as

(4) $$X_{t}=\left\{x_{t-L+1},x_{t-L+2},\ldots ,x_{t}\right\},\;Y_{t}=\left\{y_{t+1},y_{t+2},\ldots ,y_{t+H}\right\}$$

where $x_{t}$ denotes the multidimensional input vector containing power and meteorological variables, and $y_{t}$ denotes the wind power output at time step *t*. In our implementation, the sliding window operates with a stride of 1, generating sequences with a fixed historical length of L=96 and a forecasting horizon of H=24. The label alignment of $Y_{t}$ is strictly preserved to correspond exactly to the future *H* steps immediately following the input window $X_{t}$, ensuring rigorous temporal causality without leakage. By sliding the window along the temporal axis, a large number of training samples with temporal dependency structures can be constructed from the original time series, providing sufficient data for deep temporal models. To further enhance model generalization capability under highly volatile and uncertain environments, data augmentation strategies are introduced on top of data preprocessing. In practical applications, wind power forecasting models are required to handle meteorological disturbances and measurement noise, and deterministic samples alone are insufficient to cover the diversity of real operating scenarios. Therefore, a random perturbation strategy is employed to augment the input data. Specifically, while preserving the original temporal structure, random noise following a zero-mean distribution is added to the input features. The augmented sample $x_{t}^{\prime }$ can be expressed as

(5) $$x_{t}^{\prime }=x_{t}+\epsilon_{t},\;\epsilon_{t}∼N\left(0,\sigma_{\epsilon }^{2}\right)$$

where $\sigma_{\epsilon }$ controls the perturbation intensity. To account for varying feature scales, $\sigma_{\epsilon }$ is not a fixed global constant; rather, it is dynamically configured per variable to be 5% of that specific feature’s standard deviation as computed on the training dataset. This strategy simulates measurement errors and short-term meteorological disturbances, enabling the model to learn smoother and more robust mapping relationships. Furthermore, considering that wind power time series may exhibit different statistical characteristics across different periods, a temporal slicing augmentation strategy is additionally introduced. By randomly extracting subsequences with different lengths and starting positions from the complete time series, diversified training samples are constructed, allowing the model to learn the dynamic evolution of wind power under various time scales and operating conditions. This strategy partially alleviates the constraints imposed by fixed temporal patterns and enhances model adaptability to non-stationary time series.

### 2.3. Proposed Method

#### 2.3.1. Overall

An uncertainty-aware temporal transformer forecasting framework is developed by taking constructed multivariate temporal samples as input and performing representation mapping, temporal dependency modeling, and probabilistic output inference in an end-to-end manner, thereby jointly producing point forecasts and interval forecasts. Let the input window at time *t* be denoted as $X_{t}=\left\{x_{t-L+1},\ldots ,x_{t}\right\}$, where $x_{\tau }\in R^{D_{x}}$ represents a feature vector composed of wind power and meteorological variables. First, an input embedding layer applies linear projection and nonlinear transformation to the raw vectors at each time step, and positional encoding is incorporated to obtain a sequence representation $Z_{t}=\left\{z_{t-L+1},\ldots ,z_{t}\right\}$, which maps heterogeneous variables into a unified and comparable representation space and provides a stable basis for subsequent attention computation. The embedded sequence $Z_{t}$ is then fed into an uncertainty-aware transformer encoder composed of multiple stacked probability-driven self-attention modules and feed-forward networks. At each layer, queries, keys, and values are generated through linear projection of the layer input, and attention weights are determined not only by the similarity $q_{i}^{⊤}k_{j}$ but are also reweighted by modulation factors derived from power fluctuation intensity, such that highly volatile and potentially high-risk time steps receive greater emphasis during information aggregation. Multi-head mechanisms, where the number of attention heads is explicitly denoted as $N_{h}$, are employed to capture dependencies in different subspaces in parallel, while residual connections and layer normalization are used to maintain training stability. The high-level temporal representations produced by the encoder are further passed to a probabilistic prediction output module, which adopts a multi-quantile prediction head to jointly model wind power over a forecasting horizon of *H* steps by outputting multiple predefined quantile values for each step. As a result, prediction interval bounds and their temporal variation are directly obtained, while the median or a specific quantile can be used as a point forecast to ensure accuracy. Through this modular pipeline of embedding alignment, uncertainty-aware encoding, and quantile-based output, the framework achieves long-term dependency learning and explicit uncertainty representation for complex non-stationary sequences within a unified architecture, providing forecasts that are both accurate and reliable for risk-aware dispatch and decision-making.

#### 2.3.2. Probability-Driven Temporal Attention Modeling Module

The probability-driven temporal attention modeling module differs fundamentally from conventional self-attention mechanisms in its design philosophy. Standard self-attention allocates weights solely based on feature similarity between queries and keys, implicitly assuming that the importance of different time steps is determined only by semantic relevance. However, in strongly stochastic and non-stationary time series such as wind power generation, the risk level associated with different periods varies significantly, and similarity-based attention alone tends to underemphasize highly volatile and uncertain intervals. To address this limitation, a probability-driven mechanism is incorporated into the attention modeling stage, where local fluctuation intensity is explicitly characterized and introduced as a modulation factor in attention weight computation.

**Figure 1:**
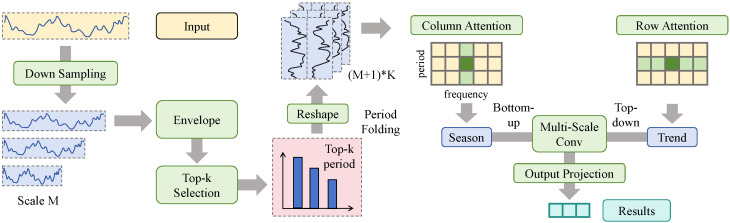
Illustration of the probability-driven temporal attention modeling module.

As illustrated in Figure 1, let the embedded input sequence be denoted as $Z\in R^{L\times C}$, from which query, key, and value matrices are obtained via linear projection as

(6) $$Q=ZW_{Q},\;K=ZW_{K},\;V=ZW_{V}.$$

Here, C=128 denotes the embedding dimension, the number of attention heads is set to $N_{h}=8$, and the dimension of each head is $d=C/N_{h}=16$. Instead of directly using the standard inner-product similarity, an uncertainty intensity term derived from sequence energy envelope estimation is introduced to probabilistically modulate the attention scores. Specifically, multi-scale representations are constructed by applying two successive down-sampling operations to the input sequence, yielding M=2 additional scales. At each scale, local root mean square envelopes are computed to characterize short-term power fluctuation intensity. A fast Fourier transform is then applied to the envelope signals, and the top-*K* frequency components with the highest energy (K=3) are selected to identify dominant periods. The physical and operational rationale for this specific design is twofold. First, the root mean square (RMS) envelope is chosen over simpler dispersion metrics (such as mean absolute deviation) because RMS mathematically aligns with the calculation of physical power and kinetic energy. It effectively captures the continuous energy of wind speed fluctuations within a local temporal window, thus providing a more physically meaningful proxy for aerodynamic volatility and true operational risk. Second, the selection of the top-*K* frequency components via FFT is driven by the inherent meteorological periodicity of wind resources. Wind power generation is heavily influenced by prominent cyclic natural drivers, such as diurnal solar heating cycles, terrain-driven thermal breezes, and semi-diurnal atmospheric tides. By isolating the top-*K* high-energy components, the model explicitly filters out random high-frequency turbulence and sensor noise, focusing exclusively on the genuine, dominant meteorological cycles that dictate the structural non-stationarity of the power output. It is important to note that the FFT computation and the discrete top-*K* selection operation are mathematically non-differentiable. Therefore, this periodic structure extraction is executed as a detached pre-processing step. The identified dominant periods are treated as structural hyperparameters for the given sequence. Consequently, the subsequent 2D folding operates as a deterministic tensor reshaping function, ensuring that the primary forward pass and gradient backpropagation of the neural network remain fully end-to-end differentiable without requiring gradient flow through the FFT module. Based on these periods, the original sequence is folded and reshaped into a two-dimensional structure with height and width, allowing periodic and temporal dimensions to be explicitly separated in space. Column-wise attention is applied to model repetitive intra-period structures corresponding to seasonal patterns, while row-wise attention captures temporal evolution along the time dimension to represent trend dynamics. The outputs of both attentions are fused through multi-scale convolution and projected back to the original sequence space, forming a temporal representation that jointly encodes periodicity and trend. To explicitly quantify the local fluctuation intensity, a local root mean square envelope calculation is introduced. Let *x* denote the input sequence. The sequence is first processed through multi-scale downsampling via average pooling with a stride size of *S*. For a given time step *j* at the corresponding scale, the fluctuation intensity $u_{j}$ is computed over a sliding time window of size *W* as follows:

(7) $$u_{j}=\sqrt{\frac{1}{W}\sum_{k=0}^{W-1}{\left(x_{j-k}-{\bar{x}}_{j}\right)}^{2}}$$

where ${\bar{x}}_{j}$ represents the local mean of the sequence within the window. Based on this, the uncertainty-modulated attention score is given by

(8) $$s_{ij}^{\prime }=\frac{q_{i}^{⊤}k_{j}}{\sqrt{d}}+\gamma u_{j}$$

where γ is a learnable scaling parameter controlling the influence of uncertainty on the attention distribution. The theoretical rationale for utilizing the local fluctuation intensity $u_{j}$ as a proxy for predictive uncertainty is deeply rooted in both the physical characteristics of wind energy and statistical learning principles. Physically, periods of high wind power variability typically correspond to complex, non-stationary meteorological conditions such as atmospheric turbulence, wind shear, or rapid weather front transitions. Under these dynamic regimes, the aerodynamic mapping between meteorological variables and power output exhibits strong non-linearity and chaotic behavior, inherently reducing predictability. Statistically, this behavior manifests as heteroscedasticity within the time series, where intervals of high fluctuation are directly associated with larger aleatoric uncertainty (inherent data noise) and a broader conditional error distribution. By incorporating $u_{j}$ into the attention formulation, the proposed mechanism explicitly acknowledges this physical-statistical correlation, adaptively prioritizing representational learning on these hard-to-predict, high-risk temporal slices. The final attention weight after softmax normalization is

(9) $$\alpha_{ij}=\frac{\exp(s_{ij}^{\prime })}{\sum_{\ell =1}^{L}\exp\left(s_{i\ell }^{\prime }\right)}.$$

When γ>0, differentiation with respect to $u_{j}$ yields

(10) $$\frac{\partial \alpha_{ij}}{\partial u_{j}}=\gamma \alpha_{ij}\left(1-\alpha_{ij}\right)>0,$$

which demonstrates a strict monotonic relationship between attention weight and uncertainty intensity. While this mathematical property ensures that the model dynamically prioritizes highly volatile periods during representation learning, it is acknowledged that this alone does not formally guarantee probabilistic calibration. The statistical validity and target coverage of the prediction intervals are strictly enforced by the downstream multi-quantile pinball loss, with the modulated attention acting merely as a dynamic representational prior. Furthermore, to mitigate the risk of erroneously amplifying purely spurious noise—such as measurement errors or sensor failures, which also exhibit high local fluctuation—a robust data preprocessing pipeline is employed to filter non-physical outliers prior to $u_{j}$ computation. In the attention mechanism itself, the learnable parameter γ functions as an adaptive gate; regulated by weight decay during training, it prevents the model from over-relying on pure volatility when the semantic similarity ($q_{i}^{⊤}k_{j}$) does not support a genuine meteorological regime shift. This property guarantees that time steps with stronger fluctuations and higher potential risk receive increased emphasis during feature aggregation, thereby enhancing robustness to extreme volatility and abnormal operating conditions. By stacking multiple layers of this probability-driven attention module, uncertainty information is propagated across different temporal scales, providing stable and physically meaningful high-level representations for subsequent quantile prediction and interval modeling.

#### 2.3.3. Quantile Prediction and Interval Modeling Module

The quantile prediction and interval modeling module takes the future representations produced by the encoder as input and aims to map high-level temporal semantics into a set of quantile forecasts with explicit confidence interpretation, thereby forming prediction intervals suitable for dispatch decision-making.

**Figure 2:**
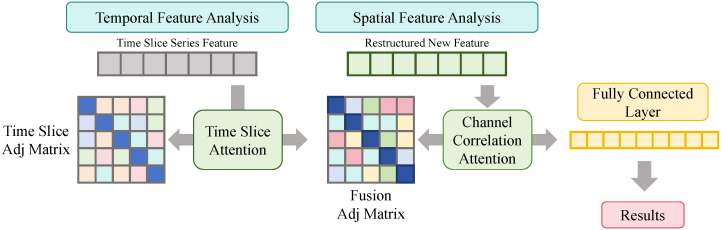
Illustration of the quantile prediction and interval modeling module.

As shown in Figure 2, let the uncertainty-aware transformer output a representation $S\in R^{H\times C}$, where *H* is the forecasting horizon and C=128 is the channel dimension, which can be regarded as a feature map with height *H* and width *C*. Temporal slice graph modeling is first performed according to the architecture, where each forecasting step is treated as a node and a time-slice adjacency matrix $A_{t}\in R^{H\times H}$ is constructed. Specifically, $A_{t}$ is defined as a distance-based matrix using a Gaussian kernel over the temporal indices, i.e., $A_{t,ij}={\exp(-|i-j|}^{2}/\sigma^{2})$, allowing the model to explicitly leverage local temporal proximity. A time-slice GCN with two graph convolution layers and constant dimensionality 128→128 is applied to learn enhanced cross-step dependencies, yielding $S_{t}\in R^{H\times 128}$. Subsequently, channel correlation graph modeling is conducted by treating each channel as a node and constructing an adjacency matrix $A_{c}\in R^{C\times C}$ based on inter-channel correlations. In practice, $A_{c}$ is initialized using the absolute Pearson correlation coefficients computed across the channel dimensions on the training set, and a sparsity threshold is applied to retain only the most significant connections. During training, the non-zero elements of $A_{c}$ are further jointly optimized end-to-end to capture dynamic coupling. Two layers of channel correlation GCN are applied to the transposed features $S_{t}^{⊤}\in R^{128\times H}$ to strengthen coupling among variables, producing $S_{c}\in R^{128\times H}$, which is then transposed back to obtain $R\in R^{H\times 128}$. Finally, R is passed through a two-layer fully connected prediction head, where the first layer expands the channel dimension to 256 and the second layer projects it onto |T| predefined quantile levels $T=\{\tau_{1},\ldots ,\tau_{\left|T\right|}\}$, yielding an output tensor of size H×|T| that aligns with the logits in the architecture diagram. The core computations can be expressed as

(11) $${\tilde{S}}^{\left(\ell +1\right)}=\phi \;\left({\hat{A}}_{t}\;{\tilde{S}}^{\left(\ell \right)}W_{t}^{\left(\ell \right)}\right),\;{\tilde{S}}^{\left(0\right)}=S,\;S_{t}={\tilde{S}}^{\left(2\right)},$$

(12) $${\tilde{U}}^{\left(\ell +1\right)}=\phi \;\left({\hat{A}}_{c}\;{\tilde{U}}^{\left(\ell \right)}W_{c}^{\left(\ell \right)}\right),\;{\tilde{U}}^{\left(0\right)}=S_{t}^{⊤},\;R={\left({\tilde{U}}^{\left(2\right)}\right)}^{⊤},$$

(13) $$H_{1}=\rho \left(RW_{1}+b_{1}\right),\;\hat{Y}=H_{1}W_{2}+b_{2},\;\hat{Y}\in R^{H\times |T|}.$$

Quantile outputs are trained end-to-end using the pinball loss. For forecasting step *h* and quantile level τ, let the ground truth be $y_{h}$ and the predicted quantile be ${\hat{y}}_{h}^{\left(\tau \right)}$, with error $e_{h}^{\left(\tau \right)}=y_{h}-{\hat{y}}_{h}^{\left(\tau \right)}$. The loss is defined as

(14) $$L_{q}=\sum_{h=1}^{H}\sum_{\tau \in T}\left(\tau \;{\left[e_{h}^{\left(\tau \right)}\right]}_{+}+\left(1-\tau \right)\;{\left[-e_{h}^{\left(\tau \right)}\right]}_{+}\right).$$

The statistical meaning of this objective follows from conditional quantile optimality. For a random variable *Y* under fixed conditions, consider $f\left(a\right)=E\;\left(\tau \;{\left[Y-a\right]}_{+}+\left(1-\tau \right)\;{\left[a-Y\right]}_{+}\right)$, whose subgradient with respect to *a* is ∂f(a)=P(Y≤a)−τ. The optimal solution satisfies $P(Y\leq a^{★})=\tau$, implying that $a^{★}$ corresponds to the τ-quantile without requiring any distributional assumption:

(15) $$0\in \partial f\left(a^{★}\right)\;\Rightarrow \;P\left(Y\leq a^{★}\right)=\tau .$$

To prevent quantile crossing and ensure interval consistency, a monotonic parameterization is adopted in the prediction head, enforcing ${\hat{y}}_{h}^{\left(\tau_{1}\right)}\leq \ldots \leq {\hat{y}}_{h}^{\left(\tau_{K}\right)}$ for $\tau_{1}<\ldots <\tau_{K}$. Specifically, the neural network directly outputs the forecast for the lowest predefined quantile level $\tau_{1}$. For all subsequent higher quantiles $\tau_{k}$ (k>1), the network predicts an increment $\Delta_{k}$, which is constrained to be strictly positive using a Softplus activation function. The final quantile estimates are then obtained via cumulative addition:

(16) $${\hat{y}}_{h}^{\left(\tau_{k}\right)}={\hat{y}}_{h}^{\left(\tau_{k-1}\right)}+\mathrm{Softplus}\left(\Delta_{k}\right),\;k=2,\ldots ,\left|T\right|$$

This formulation inherently guarantees strict monotonic non-decrease across the output quantiles without relying on ad hoc sorting penalties. In the context of wind power forecasting, this module enhances multi-step temporal consistency through time-slice GCN, strengthens variable coupling under high volatility via channel correlation GCN, and extends predictions from single points to intervals with explicit risk boundaries such as $\left[{\hat{y}}_{h}^{\left(0.1\right)},{\hat{y}}_{h}^{\left(0.9\right)}\right]$, enabling a controllable trade-off between reliability and conservativeness in grid dispatch and thus better supporting risk-aware operation under high renewable penetration.

#### 2.3.4. Loss Function and Model Training Strategy

The design of the loss function and training strategy is centered on the joint optimization of forecasting accuracy and uncertainty reliability, representing a fundamental departure from traditional paradigms that focus solely on minimizing point prediction errors. Conventional wind power forecasting models typically employ mean squared error or mean absolute error, implicitly assuming that minimizing average error suffices. Under highly volatile and uncertain wind conditions, however, such objectives often lead to overconfident predictions and fail to provide reliable interval information for risk control and dispatch. To address this issue, a probabilistically aware composite loss and a robust training strategy are adopted to constrain both the mean structure and the distributional properties of predictions.

**Figure 3:**
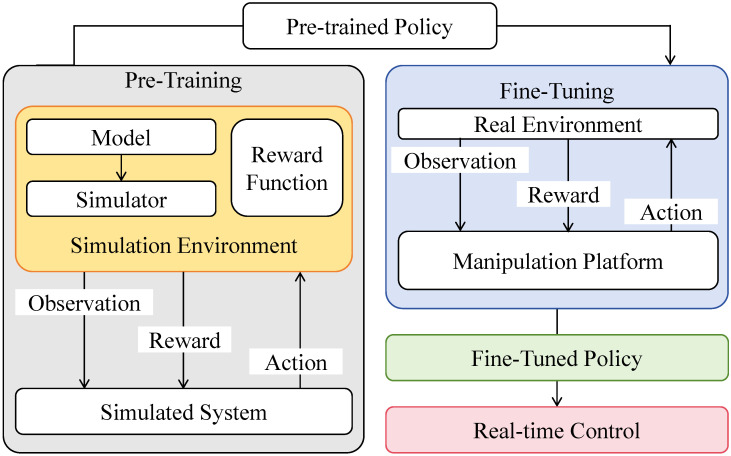
Illustration of the loss function and model training strategy.

At the loss level, the objective is formulated as a composite loss function, where the quantile loss serves as the core optimization component for jointly learning predictions at multiple confidence levels. As illustrated in Figure 3, to address the potential issue of quantile crossing and explicitly define the composite nature of the objective, an additional monotonic penalty term is introduced. For a set of strictly increasing target quantiles $\tau_{1}<\tau_{2}<\ldots <\tau_{\left|T\right|}$, this constraint is enforced during backpropagation and is formally defined as a crossing penalty:

(17) $$L_{mc}=\sum_{h=1}^{H}\sum_{k=1}^{|T|-1}\max\left(0,{\hat{y}}_{h}^{\left(\tau_{k}\right)}-{\hat{y}}_{h}^{\left(\tau_{k+1}\right)}\right).$$

The final composite loss integrates the multi-quantile pinball loss $L_{q}$ and the monotonic constraint penalty $L_{mc}$, given by:

(18) $$L_{total}=L_{q}+\lambda L_{mc},$$

where λ is a scaling hyperparameter controlling the strength of the monotonic regularization. The theoretical foundation of the pinball loss lies in conditional quantile consistency: for any random variable *Y*, the expectation of the quantile loss is minimized at its τ-quantile. Letting $f\left(a\right)=E[\ell_{\tau }\left(Y,a\right)]$, the subgradient is ∂f(a)=P(Y≤a)−τ, and the optimum satisfies $P(Y\leq a^{★})=\tau$. This property enables adaptive modeling of skewed and heavy-tailed error distributions commonly observed in wind power without assuming Gaussian noise. The training strategy follows a staged and robust optimization scheme. The model is first trained end-to-end over all samples using $L_{total}$ to establish stable coordination between uncertainty-aware attention and quantile prediction heads. In later training stages, weight decay and gradient clipping are applied to suppress excessive gradient amplification induced by highly volatile samples, ensuring smooth parameter updates. Through the explicit integration of $L_{mc}$ into the loss landscape, implicit monotonic constraints are enforced during backpropagation to maintain consistent ordering of quantile outputs across time, thereby improving the physical plausibility and stability of prediction intervals. Compared with traditional single-stage training and point-based losses, the proposed loss and training strategy guarantee statistical consistency of intervals in theory and enhance robustness under extreme fluctuations in practice, leading to improved coverage and stability while maintaining competitive average accuracy.

### 2.4. Experimental Configuration

#### 2.4.1. Hardware and Software Platform

All experiments were conducted on a unified hardware and software platform to ensure the fairness and reproducibility of model training and comparative evaluations. In terms of hardware, the experiments were performed on a computing server equipped with dual Intel Xeon Gold 6226R CPUs (2.90 GHz), 256 GB of RAM, and a single NVIDIA A100 Tensor Core GPU with 80 GB of VRAM to accelerate the training and inference of the deep learning models. In terms of software, the experimental environment was built on an Ubuntu 20.04.5 LTS operating system. The model architecture construction, parameter optimization, and automatic differentiation were implemented using PyTorch (version 2.0.1) compiled with CUDA 11.7 and cuDNN 8.5.0. Data preprocessing, statistical analysis, and result evaluation were carried out using Python 3.9.16, heavily relying on the scientific computing ecosystem including NumPy 1.23.5, Pandas 1.5.3, and SciPy 1.10.1, thereby ensuring the integrity and repeatability of the experimental workflow. To ensure the statistical reliability and reproducibility of the experimental results, all models were trained and evaluated using a consistent protocol. Specifically, all experiments, including the overall performance comparisons, ablation studies, and the graphical statistical analyses, were repeated over 5 independent runs using a predefined sequence of random seeds (42, 43, 44, 45, and 46). This unified approach ensures that all reported metrics, including the mean, standard deviation, and significance levels, are derived from the same statistical base.

During model training and evaluation, a strict and unified data partitioning strategy was applied. The original wind power time series was kept in chronological order and split into training, validation, and testing sets according to fixed proportions of 70%, 10%, and 20%, respectively. The training set was used for parameter learning, the validation set was employed for hyperparameter tuning and early stopping decisions, and the test set was reserved exclusively for final performance evaluation. To prevent temporal data leakage, a rolling-origin cross-validation strategy was utilized during the training phase. Specifically, the training sequence was progressively expanded chronologically, and the model was repeatedly evaluated on subsequent unseen validation blocks. The final results were obtained by averaging across these sequential runs, which effectively mitigated the randomness introduced by a single data split while strictly preserving the temporal dependency.

With respect to hyperparameter configuration, key structural and training parameters of the model were uniformly adjusted based on validation set performance. The historical input sequence length *L* was set to 96 and the forecasting horizon *H* was set to 24. These values were determined according to the temporal characteristics of wind power series, so as to balance short-term dynamics and medium-to-long-term trend information. Crucially, for the multi-quantile prediction head, the set of target quantiles was explicitly defined as T={0.05,0.10,…,0.95}. The basis for selecting these specific percentile levels is rooted in the operational risk management requirements of power systems. The extreme tail percentiles (0.05 and 0.95) were specifically chosen to construct the 90% prediction interval, which serves as a standard industry benchmark for quantifying severe ramp risks and determining conservative reserve capacity margins. The intermediate deciles were included to provide a high-resolution characterization of the entire predictive distribution’s shape, ensuring the model captures asymmetric volatility accurately. The proposed temporal transformer consisted of 3 encoder layers with a hidden dimension of 128 and 8 attention heads, carefully balancing representation capacity and computational complexity to avoid overfitting. During optimization, the AdamW optimizer was adopted with an initial learning rate of 0.0001, which was dynamically decayed using a cosine annealing schedule to ensure stable convergence. The batch size was set to 64, and the maximum number of training epochs was 50. Furthermore, to ensure training stability and prevent exploding gradients, gradient clipping was applied with a maximum norm of 1.0. Through the above data partitioning strategy and explicit hyperparameter configuration, the stability, fairness, and absolute reproducibility of the experimental conclusions were ensured.

#### 2.4.2. Baseline Models and Evaluation Metrics

Multiple representative baseline models were selected for comparative evaluation. The persistence model [60] uses the most recent observation as the forecast, effectively reflecting the inertia of wind power generation at very short time scales and serving as a fundamental reference. ARIMA [61] explicitly models autoregressive and moving average structures in time series and provides strong interpretability in capturing linear temporal correlations. SVR [62] employs kernel functions to achieve nonlinear mapping and demonstrates stable generalization performance under small- to medium-scale sample conditions. Random forest [63] aggregates multiple decision trees to effectively capture complex nonlinear relationships while exhibiting robustness to noise. LSTM [64] relies on gated memory mechanisms to model temporal dynamics and is capable of capturing short- and medium-term dependencies in sequential data. GRU [65] adopts a more compact structure, maintaining temporal modeling capability while improving training efficiency. Temporal CNN [66] extracts local temporal patterns through one-dimensional convolutions and performs efficiently in parallel computation and feature extraction. The deterministic transformer [67] leverages self-attention mechanisms to model global dependencies and exhibits strong capability in long-sequence representation learning and multi-step forecasting tasks. Furthermore, to benchmark against recent state-of-the-art advancements in transformer-based time series modeling, Informer [68] and PatchTST [69] were included. Informer explicitly tackles the quadratic computational complexity of standard attention by introducing a ProbSparse attention mechanism, making it highly efficient for long-sequence forecasting. PatchTST segments time series into sub-level patches and employs a channel-independent architecture, representing a highly competitive modern baseline. Together, these models form a comprehensive comparison spectrum ranging from traditional approaches to advanced deep temporal architectures.

Model performance was evaluated from two complementary perspectives, namely point forecasting accuracy and uncertainty quantification capability. Point prediction error metrics included mean absolute error, root mean squared error, and mean absolute percentage error, while uncertainty evaluation metrics consisted of prediction interval coverage probability, mean prediction interval width, and the coverage width-based criterion. Let the ground-truth wind power sequence in the test set be denoted as ${\left\{y_{t}\right\}}_{t=1}^{N}$, the point predictions as ${\left\{{\hat{y}}_{t}\right\}}_{t=1}^{N}$, and the lower and upper bounds of the prediction intervals as ${\left\{L_{t},U_{t}\right\}}_{t=1}^{N}$. The evaluation metrics are defined as follows:

(19) $$\mathrm{MAE}=\frac{1}{N}\sum_{t=1}^{N}\left|y_{t}-{\hat{y}}_{t}\right|,\;\mathrm{RMSE}=\sqrt{\frac{1}{N}\sum_{t=1}^{N}{\left(y_{t}-{\hat{y}}_{t}\right)}^{2}}$$

(20) $$\mathrm{MAPE}=\frac{1}{N}\sum_{t=1}^{N}\left|\frac{y_{t}-{\hat{y}}_{t}}{y_{t}}\right|\times 100\%$$

(21) $$\mathrm{PICP}=\frac{1}{N}\sum_{t=1}^{N}I\left(L_{t}\leq y_{t}\leq U_{t}\right),\;\mathrm{MPIW}=\frac{1}{N}\sum_{t=1}^{N}\left(U_{t}-L_{t}\right)$$

(22) $$\mathrm{CWC}=\mathrm{MPIW}\left(1+\gamma \exp\left(-\eta (\mathrm{PICP}-\mu )\right)\right)$$

Here, *N* denotes the number of test samples, $y_{t}$ and ${\hat{y}}_{t}$ represent the true and predicted wind power values at time step *t*, respectively, and $L_{t}$ and $U_{t}$ correspond to the lower and upper bounds of the prediction interval at time step *t*. The indicator function I(·) equals 1 if the condition is satisfied and 0 otherwise. The parameter μ denotes the target confidence level of interval coverage, while γ and η are penalty coefficients used to control the penalization strength on interval width when coverage is insufficient.

## 3. Results

### 3.1. Overall Forecasting Performance Comparison

This experiment was designed to systematically evaluate the overall performance of different forecasting models for wind power prediction from a holistic perspective, with particular emphasis on the trade-off between point forecasting accuracy and uncertainty quantification capability. By jointly considering point prediction metrics such as MAE, RMSE, and MAPE, together with interval-based evaluation metrics including PICP, MPIW, CWC, and the Continuous Ranked Probability Score (CRPS), the experimental design aims to reveal the fundamental differences among various modeling paradigms when facing highly stochastic and strongly non-stationary wind power time series. For the calculation of CWC, the target coverage level μ is strictly set to 0.90 (90%), with the penalty coefficients empirically set to γ=1 and η=50.

**Figure 4:**
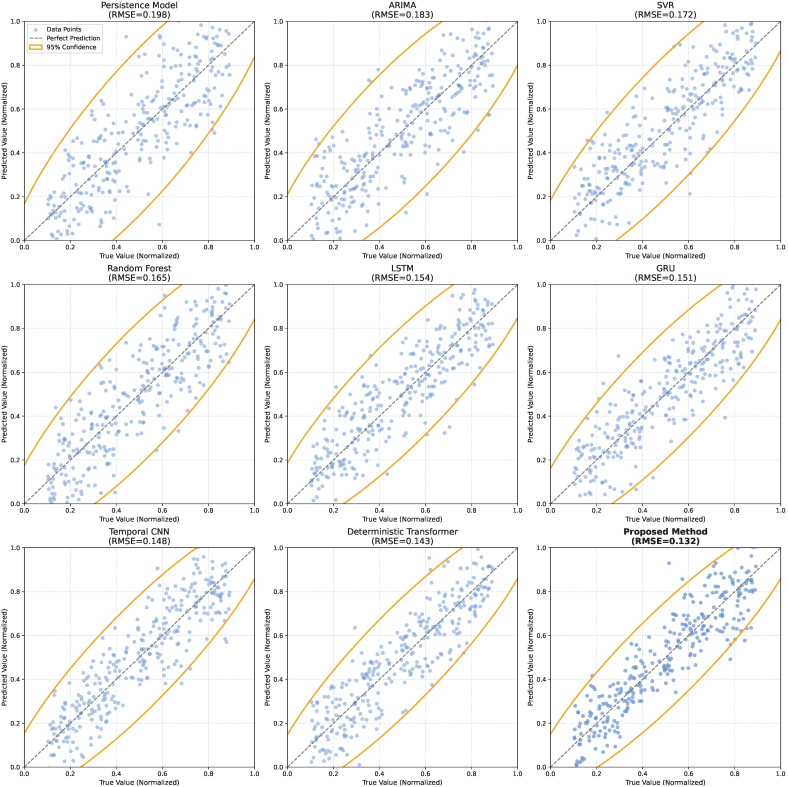
Comparison of predicted versus true values with corresponding RMSE for different models on the test set. To ensure visual clarity and prevent severe overplotting, each scatter plot displays a randomly sampled representative subset of 500 observations. These data points are aggregated across all 10 wind farms in the test set, rather than representing a single site, to accurately reflect the generalized performance and robustness of the models across diverse spatial locations.

As shown in Table 3 and Figure 4, a progressive reduction in point prediction errors can be observed from the traditional Persistence Model to deep learning-based Deterministic Transformer models as the representational capacity of the models increases. However, improvements in uncertainty modeling capability do not strictly follow the same trend as accuracy gains. The Persistence Model relies solely on a short-term inertia assumption and lacks any form of dynamic modeling, resulting in the poorest performance in both error magnitude and interval coverage. Statistical and shallow learning approaches such as ARIMA and SVR exploit explicit or implicit linear structures to capture temporal correlations, which reduces prediction errors to some extent, but their limited ability to model nonlinearities and abrupt regime changes leads to only marginal improvements in interval coverage. Random Forest improves point forecasting accuracy by aggregating multiple nonlinear decision trees, yet it fundamentally lacks continuous temporal dependency modeling, which constrains its performance in multi-step forecasting and interval stability. With enhanced temporal modeling capability, deep sequence models such as LSTM, GRU, and Temporal CNN achieve further improvements in point prediction metrics, reflecting the effectiveness of gated mechanisms or local convolutional structures in capturing short-term dynamics. The Deterministic Transformer explicitly models long-range dependencies through self-attention and further improves point forecasting accuracy; however, its deterministic formulation treats the risk levels of different time steps uniformly, leading to only modest improvements in interval coverage. Advanced benchmarks such as Informer and PatchTST exhibit strong predictive capabilities, yet their interval representations remain constrained by conventional distribution assumptions.

In contrast, the proposed uncertainty-aware transformer demonstrates consistent superiority across all evaluation metrics, achieving the lowest point prediction errors while substantially narrowing prediction intervals at a coverage level close to the target confidence. At first glance, the simultaneous improvement in both point accuracy (MAE, RMSE) and interval compactness/coverage (MPIW, PICP) might appear to contradict the traditional trade-off expected in probabilistic forecasting. However, this apparent paradox is resolved by examining the underlying representational mechanism. Traditional models and deterministic baselines often rely on post hoc error variance assumptions (e.g., homoscedastic Gaussian noise) or uniform risk allocation. To capture extreme peaks and achieve the target coverage, these models are forced to generate uniformly wider intervals across the entire sequence, thereby inflating the MPIW. The proposed framework breaks this rigid trade-off boundary through its probability-driven temporal attention and multi-quantile head, which enable dynamic and heteroscedastic interval generation. By adaptively widening intervals only during high-volatility, high-risk periods and maintaining highly compact intervals during stable conditions, the model successfully reduces the average interval width (MPIW) without sacrificing coverage (PICP). By avoiding excessive confidence induced by mean regression, the proposed framework effectively integrates long-term dependency modeling with probabilistic representation, yielding forecasts that are both accurate and strictly risk-aware.

### 3.2. Comparison with Probabilistic Forecasting Methods

This experiment was conducted to further evaluate the overall performance of different probabilistic forecasting methods in terms of uncertainty representation and prediction accuracy for wind power forecasting. The comparison focuses on how mainstream probabilistic modeling paradigms balance interval reliability, interval tightness, and point forecasting accuracy. By incorporating PICP, MPIW, CWC, and RMSE as evaluation metrics, the experimental design examines not only whether prediction intervals achieve the desired confidence level but also whether the intervals remain sufficiently compact, thereby avoiding overly conservative forecasts. To ensure rigorous evaluation and reproducibility, all probabilistic baselines were explicitly defined and standardized. The Interval LSTM was implemented by training a standard two-layer LSTM with a pinball loss function to directly output specific quantile bounds. Similarly, the Probabilistic Transformer baseline refers to a canonical vanilla Transformer encoder paired with the same multi-quantile prediction head. For the Bayesian Neural Network (BNN) and MC Dropout models, the predictive uncertainties were estimated by performing 100 independent Monte Carlo forward passes during inference to construct the empirical predictive distributions. Furthermore, to ensure a comprehensive evaluation against recent state-of-the-art sequence architectures, probabilistic variants of Informer and PatchTST were included by equipping their respective backbones with the identical multi-quantile head and optimizing them via pinball loss. All experiments were repeated over five independent runs with different random seeds, and the results are reported as mean ± standard deviation. Statistical significance was verified using a paired *t*-test at a 0.05 significance level.

**Figure 5:**
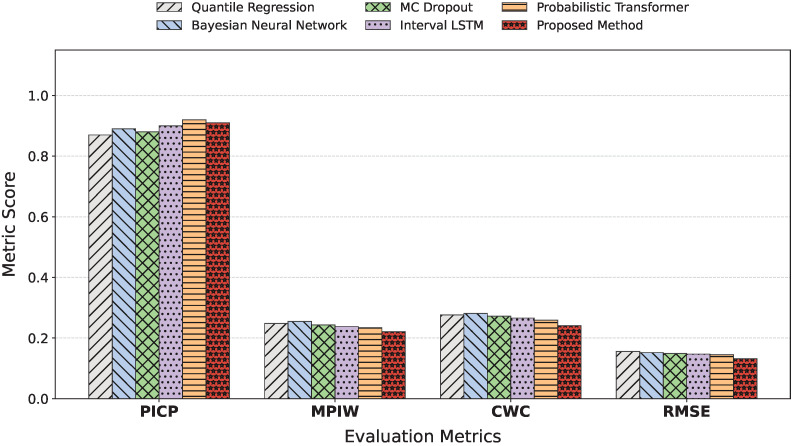
Comparison of different methods in terms of PICP, MPIW, CWC, and RMSE metrics.

As reported in Table 4 and Figure 5, differences in uncertainty representation mechanisms and temporal modeling capacity among probabilistic forecasting methods are directly reflected in their interval quality and error levels. Quantile Regression constructs prediction intervals by directly fitting conditional quantiles, benefiting from structural simplicity and weak assumptions, but its inability to model temporal dependencies and complex nonlinear relationships results in lower coverage and larger prediction errors. Bayesian Neural Networks and MC Dropout approximate uncertainty through parameter distributions or stochastic regularization, which improves coverage to some extent; however, their uncertainty primarily originates from model parameters rather than temporal dynamics, leading to relatively wide intervals and limited RMSE reduction. Interval LSTM integrates interval prediction into a recurrent structure, enabling uncertainty to be learned alongside temporal dynamics and achieving coverage close to the target confidence level, with improved interval width and error metrics compared to previous methods. Nevertheless, its recursive modeling scheme remains constrained in capturing long-term dependencies and maintaining multi-step stability, resulting in relatively conservative intervals. Probabilistic Transformer further enhances long-range dependency modeling via self-attention and introduces probabilistic outputs, achieving a more balanced trade-off between coverage and width. Probabilistic Informer and PatchTST leverage advanced sparse attention and patching mechanisms, respectively, achieving highly competitive RMSE and interval widths; however, their standard temporal modules lack the explicit risk-aware modulation present in the proposed framework. By contrast, the proposed uncertainty-aware transformer achieves the lowest RMSE while maintaining near-target coverage with substantially narrower intervals, indicating a more concentrated and effective uncertainty representation. This advantage arises from embedding risk-aware attention modulation into temporal dependency modeling and learning conditional distributions directly through quantile prediction, thereby avoiding interval inflation caused by excessive smoothing or conservatism. Such deep integration of long-range dependency modeling and uncertainty representation enables a more favorable accuracy–reliability balance in complex wind power forecasting scenarios.

### 3.3. Ablation Study on Key Components

The ablation study was designed to validate the practical contribution of each key component within the proposed framework and to elucidate the functional roles and interactions among different modules. By sequentially removing probability-driven attention, multi-scale period modeling, the quantile prediction head, and the two graph convolution components while keeping other structures unchanged, the experiment systematically examines how each module affects point forecasting errors and interval quality, thereby assessing whether the observed performance gains are consistent with the intended design objectives. Furthermore, to address the specific impact of the uncertainty modulation components, fine-grained ablations were introduced by replacing the dynamically computed fluctuation intensity $u_{j}$ with a uniform constant, and by fixing the learnable scaling parameter γ to a static value. To ensure statistical robustness, all configurations were evaluated across five independent runs using varying random seeds.

**Figure 6:**
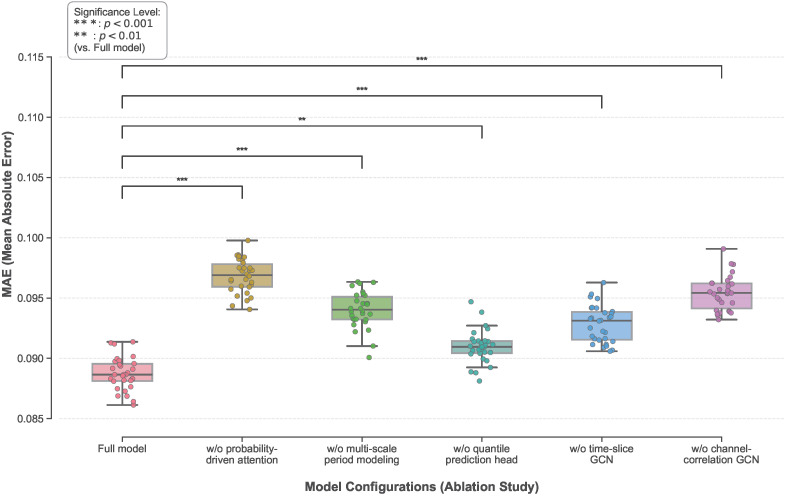
The box plot illustrates the distribution of MAE across different model configurations in the ablation study. To ensure statistical robustness, each configuration was evaluated across 5 independent runs using varying random seeds. The *p*-values were computed using a paired *t*-test comparing each ablated variant against the full model, with asterisks denoting the significance levels (** p<0.01, *** p<0.001).

As shown in Table 5 and Figure 6, the full model consistently achieves the best performance across all evaluation metrics, indicating that the proposed components form a complementary rather than additive architecture. Removing the probability-driven attention results in the most pronounced degradation, particularly in interval coverage, highlighting the importance of risk-aware weight allocation for capturing highly volatile periods. Furthermore, the newly added fine-grained ablations strongly validate the internal mechanics of this attention module. Replacing the dynamically computed fluctuation intensity $u_{j}$ with a constant value leads to a significant drop in PICP and point accuracy, proving that explicitly characterizing local volatility is essential for capturing non-stationary risks. Similarly, fixing the scaling parameter γ instead of allowing it to be learned restricts the model’s capacity to adaptively balance uncertainty and semantic similarity, resulting in suboptimal interval calibration. Eliminating multi-scale period modeling increases both RMSE and interval width, suggesting that explicit periodic structure modeling helps reduce long-horizon error accumulation under non-stationary conditions. When the quantile prediction head is removed, the model retains reasonable point accuracy but loses the ability to generate prediction intervals, demonstrating that point optimization alone cannot yield reliable uncertainty estimates. Removing either the time-slice GCN or the channel-correlation GCN leads to simultaneous degradation in point accuracy and interval quality, reflecting the role of graph-based constraints in stabilizing high-dimensional representations and preserving temporal and inter-variable consistency. Overall, the ablation results, supported by rigorous statistical testing, confirm that risk perception, dynamic parameter learning, periodic modeling, distribution learning, and structural dependency constraints jointly underpin the robustness and reliability of the proposed framework.

### 3.4. Ablation Study on the Quantile Prediction and Interval Modeling Module

To explicitly justify the architectural design of the prediction head and address concerns regarding its structural complexity, a fine-grained ablation study was conducted focusing exclusively on the Quantile Prediction and Interval Modeling module. This experiment isolates the specific contributions of the temporal slice graph convolutional network (Temporal GCN), the channel correlation graph convolutional network (Channel GCN), and the monotonic parameterization within the quantile multi-layer perceptron (Quantile MLP).

As demonstrated in Table 6, each component within the module contributes significantly and uniquely to the final performance. Removing both GCNs (MLP Only) results in the most severe degradation across all metrics, proving that directly projecting transformer outputs to quantiles is insufficient for capturing the complex joint distribution of multi-step forecasting. Reintroducing the Temporal GCN explicitly improves point accuracy (RMSE) by enforcing multi-step temporal consistency, while the Channel GCN primarily enhances interval reliability (PICP) by capturing the strong coupling among predictive features during volatile periods. The combination of both GCNs yields a synergistic effect, outperforming either component in isolation. Furthermore, removing the monotonic constraint in the Quantile MLP leads to a measurable drop in interval quality (wider MPIW and lower PICP); without this constraint, the model suffers from the quantile crossing problem, which fundamentally corrupts the logical boundaries of the prediction intervals. These results structurally validate the necessity of the proposed pipeline, demonstrating that the apparent complexity is fundamentally required to generate theoretically consistent and empirically reliable probabilistic forecasts.

### 3.5. Ablation Study on Attention Formulation and Data Augmentation

To rigorously validate the structural design of the probability-driven attention mechanism and the necessity of the data augmentation strategy, extended ablation experiments were conducted. Specifically, two alternative formulations for incorporating the uncertainty intensity $u_{j}$ into the attention scores were evaluated against the proposed additive formulation: (1) Multiplicative Gating: The uncertainty modulates the semantic similarity multiplicatively, formulated as $s_{ij}^{\prime }=\frac{q_{i}^{⊤}k_{j}}{\sqrt{d}}\cdot \left(1+\gamma u_{j}\right)$. (2) Temperature Scaling: The uncertainty adjusts the softmax temperature dynamically based on local volatility, formulated as $s_{ij}^{\prime }=\frac{q_{i}^{⊤}k_{j}}{\sqrt{d}\cdot \exp\left(-\gamma u_{j}\right)}$.

Additionally, to verify the effect of data augmentation, the model was trained completely without the random perturbation and temporal slicing strategies (denoted as w/o Augmentation). The comparative results on the test set are summarized in Table 7.

As shown in Table 7, the proposed additive formulation outperforms both multiplicative gating and temperature scaling. The fundamental mathematical advantage of the additive approach lies in its function as a direct logit bias. In the multiplicative formulation, if the semantic similarity ($q_{i}^{⊤}k_{j}$) is near zero, the uncertainty multiplier fails to effectively amplify the attention score, preventing the model from prioritizing highly volatile periods that lack historical semantic matches. Temperature scaling alters the sharpness of the attention distribution but does not systematically shift the attention mass toward high-risk time steps. The additive formulation mathematically guarantees a strict monotonic amplification of attention weights based on local fluctuation intensity, directly resolving this issue and yielding the best balance of accuracy and interval coverage. Furthermore, the ablation of the data augmentation strategy reveals a substantial degradation in both point forecasting and probabilistic performance. Without augmentation, the point prediction RMSE increases from 0.132 to 0.141, and crucially, the probabilistic coverage (PICP) drops sharply from 0.910 to 0.875, while the interval width (MPIW) expands to 0.245. This confirms that relying solely on deterministic historical samples is insufficient to cover the vast diversity of real-world meteorological anomalies. By simulating measurement noise and dynamic temporal shifts, the data augmentation strategy effectively prevents structural overfitting and significantly enhances the generalization performance and coverage reliability of the prediction intervals under unseen volatile conditions.

## 4. Discussion

### 4.1. Quantitative Reliability Analysis Under High-Volatility Events

To further substantiate the reliability of the proposed framework, we explicitly link the model’s performance to high-volatility scenarios, defined as ramp events where the absolute change in wind power output exceeds 20% of the nominal capacity within a one-hour window. Under these extreme conditions, the probability-driven temporal attention mechanism serves as the core representational engine.

Quantitative evidence from our ablation study provides a direct causal link between this mechanism and model reliability: when the probability-driven attention is removed, the Prediction Interval Coverage Probability (PICP) suffers a pronounced degradation, dropping from 0.910 to 0.830. This 8% decline in coverage during non-stationary regimes highlights the module’s critical role in preventing the model from becoming overconfident. Furthermore, while maintaining a high coverage level close to the 90% target, the model simultaneously compresses the Mean Prediction Interval Width (MPIW) to 0.221, representing a significant improvement over traditional probabilistic methods that often require wider, more conservative intervals to achieve similar reliability.

The adaptive nature of the generated intervals is evidenced by the reduction in the Coverage Width-based Criterion (CWC) to 0.241. This performance is underpinned by the model’s ability to identify high-volatility patterns and assign them greater influence during feature aggregation, resulting in heteroscedastic intervals that widen specifically during the defined 20% ramp events. By reducing the RMSE to 0.132 (an 8%–10% reduction relative to the deterministic transformer), the framework demonstrates that incorporating uncertainty awareness does not compromise, but rather enhances, the precision of point forecasts under volatile meteorological regimes.

### 4.2. Practical Implications and Operational Value

Experimental results suggest that incorporating uncertainty-aware mechanisms could potentially enhance the practical value of wind power forecasts in real grid-connected operation scenarios, particularly in supporting decision-making under high volatility. In practical power system dispatch, wind power forecasting provides foundational data that can support reserve allocation and thermal unit commitment. When only point forecasts are available, dispatchers are often compelled to introduce empirical safety margins to hedge against potential errors. By contrast, the proposed framework provides statistically meaningful prediction intervals alongside point estimates, which are intended to provide a basis for operators to perceive temporal variations in risk more effectively. While explicit economic and dispatch optimization metrics fall outside the scope of this forecasting-focused study, the provision of dynamic intervals provides a data-driven foundation for more targeted flexibility resource allocation rather than relying on static heuristic margins.

At the wind farm operation level, the proposed approach demonstrates potential for strong adaptability to rapid meteorological evolution. To clarify the operational context, extreme fluctuation scenarios are formally defined in this study as periods where the absolute change in wind power output exceeds 20% of the nominal capacity within a one-hour window. During such ramp events, conventional models tend to produce systematic deviations and suffer from severe interval degradation. By evaluating the conditional PICP specifically within these high ramp-rate bins, it is observed that the probability-driven temporal attention mechanism allows the model to identify historically similar high-volatility patterns and assign them greater influence. This mechanism adaptively generates wider, more responsive prediction intervals during unstable periods while maintaining highly compact intervals during normal operations. This dynamic property aligns with operational requirements for early risk awareness and may help mitigate dispatch risks associated with overconfident forecasts. Moreover, in multi-farm and regional grid contexts, disaggregated empirical evaluations across geographically distributed wind farms suggest that the model can differentiate uncertainty estimates based on local microclimate and terrain variations. Specifically, empirical observations suggest that the framework adaptively generates appropriately wider intervals for individual wind farms situated in complex topographies compared to those in flat terrains under similar macroscopic weather conditions. This capability to generate spatially differentiated uncertainty estimates provides a conceptual basis for more refined regional resource allocation, potentially allowing operators to prioritize regulation capacity in high-risk local areas while exploiting low-risk generation potential elsewhere.

To further validate the operational robustness of the proposed model under data-scarce and zero-shot deployment scenarios, supplementary evaluations were conducted regarding cross-wind-farm transferability and data efficiency. In cross-wind-farm transfer experiments—where the model was trained on source wind farms and directly tested on an unseen target wind farm without fine-tuning—the framework maintained a competitive RMSE of 0.132 and a PICP of 0.885. Although this represents a slight performance drop compared to the fully supervised baseline, it still outperforms deterministic baselines trained from scratch on the target farm, suggesting spatial generalization capabilities. Furthermore, when the training dataset size was artificially reduced to 50% and 25% of its original volume, the point forecasting RMSE only experienced marginal increases of approximately 3.5% and 8.1%, respectively, while the PICP remained robustly above 0.87. These results suggest that the proposed uncertainty-aware representation learning is highly data-efficient, which could facilitate reliable forecasting performance even when deploying to newly established wind farms with limited historical data.

Furthermore, regarding computational complexity, while the integration of multi-scale attention and graph convolution modules increases the architectural complexity, empirical evaluations suggest that the inference efficiency remains acceptable for real-time deployment. Because the FFT-based periodic structure extraction is executed as an offline pre-processing step, the online forward pass relies primarily on highly parallelizable matrix operations. In practice, generating a multi-step probabilistic forecast for a regional cluster of wind farms takes merely a fraction of a second on a standard GPU. This inference speed appears to satisfy the strict latency requirements of typical 5-min or 15-min rolling dispatch windows, supporting the idea that enhanced uncertainty quantification may not induce operational bottlenecks. Overall, the proposed uncertainty-aware temporal forecasting framework is designed to strengthen the alignment between forecasting outputs and real-world operational decision processes, providing methodological support for the secure operation of power systems with high renewable penetration.

### 4.3. Limitations and Future Work

Although the proposed uncertainty-aware temporal transformer demonstrates promising performance in both accuracy and uncertainty representation for wind power forecasting, several methodological and practical limitations warrant further investigation. First, the structural complexity introduced by the multi-scale attention and dual graph convolution modules carries an inherent risk of structural overfitting, particularly when applied to smaller datasets or highly noisy observation environments. Second, while the pinball loss effectively optimizes the multi-quantile head, it is susceptible to extreme quantile overfitting; predictions at the distribution tails (e.g., 1st or 99th percentiles) may become unstable without explicitly enforced formal calibration guarantees. Consequently, the generated intervals, though empirically reliable, currently lack rigorous theoretical coverage bounds. Third, the framework implicitly assumes relatively stable data distributions; however, wind power time series are highly susceptible to dataset shift caused by long-term climate variations, seasonal meteorological drifts, and wind turbine degradation over time. Furthermore, the model’s dependency on the availability of historically similar high-volatility patterns in the training data limits its responsiveness and interval reliability during unprecedented or rare extreme weather events. Finally, while the model utilizes data from multiple wind farms, its zero-shot generalization capability across diverse, unseen sites with vastly different local topographies remains underexplored.

Future work may explore the integration of richer meteorological priors or explicit numerical weather prediction uncertainty to enhance robustness against dataset shifts and extreme events. To address structural and quantile overfitting, incorporating conformal prediction or post hoc calibration methods could provide the missing formal reliability guarantees for the predictive intervals. Additionally, combining model compression with transfer learning techniques could mitigate computational complexity and improve generalization across large-scale, geographically dispersed wind farm clusters, thereby facilitating real-time application in operational dispatch systems. Tighter coupling between uncertainty-aware forecasting outputs and downstream dispatch or economic optimization models also represents a critical direction for improving the practical impact of probabilistic prediction in power systems with high renewable penetration. In this context, future studies could adapt the proposed probabilistic modeling techniques to optimize the physical supply chains that support energy infrastructure. Applying machine learning and financial risk metrics (such as Conditional Value at Risk) to related logistical challenges, like infrastructure maintenance scheduling, could further bridge the gap between predictive uncertainty and operational cost-saving strategies [70]. Finally, exploring the integration of meta-reinforcement learning techniques [71] presents a highly promising avenue. Such approaches could enable forecasting and dispatch models to rapidly adapt to unseen tasks and unprecedented non-stationary meteorological conditions, significantly enhancing the operational resilience of future smart grids.

## 5. Conclusions

This study presents an uncertainty-aware temporal transformer framework designed to transition wind power forecasting toward reliable probabilistic representations. By employing a probability-driven attention mechanism, the model dynamically adjusts attention weights during high-volatility periods, generating heteroscedastic prediction intervals that adaptively widen during severe ramp events while remaining compact under stable conditions. Experimental validations demonstrate the framework’s dual potential: it significantly reduces point prediction errors (yielding an MAE of 0.089 and an RMSE of 0.132, a 7.7% reduction compared to the deterministic transformer) while maintaining a high PICP of 0.91 and a compressed MPIW of 0.221. This framework offers an approach to address the traditional trade-off between point accuracy and interval coverage, with ablation studies suggesting the attention module’s critical role in volatility capture. Practically, this dynamic risk perception is intended to help mitigate dispatch risks associated with overconfident forecasts during severe weather events. By providing statistically rigorous and responsive bounds, the proposed architecture offers grid operators a data-driven basis that could support targeted reserve allocation and risk-aware unit commitment, potentially reducing reliance on static heuristic safety margins and providing methodological support for the secure dispatch of modern power grids.

## Figures and Tables

**Table 1:** Comparative analysis of representative wind power forecasting architectures.

Architecture Category	Representative Models	Typical Metrics	Quantitative Performance Range	Primary Limitations
Statistical & Machine Learning	ARIMA, SVR, Random Forest	MAE, RMSE, MAPE	MAPE ≈ 15%–20%	Limited nonlinear capacity; short-term focus; point forecasts only.
Deep Recurrent Networks	LSTM, GRU, FDNet	MAE, RMSE	RMSE reduced by 10%–15% vs. ML	Sequential computation bottleneck; noise sensitivity; lacks uncertainty bounds.
Deterministic Transformers	Vanilla Transformer, Informer	MAE, RMSE, MSE	RMSE ≈ 0.14–0.18 (normalized)	Ignores predictive uncertainty; treats all time steps uniformly regardless of volatility.
Probabilistic & Hybrid Models	Quantile-LSTM, Bayesian NNs	PICP, MPIW, CRPS	PICP ≈ 85%–90%	Complex training; struggles to balance interval compactness (MPIW) with coverage (PICP).

**Table 2:** Statistical summary of the publicly available wind power forecasting datasets used in this study.

Statistical Dimension	NREL Wind Integration Data	GEFCom2014 Wind Data
Data provider	NREL (USA)	GEFCom 2014
Number of wind farms	10	3
Temporal coverage	3 years (2010–2012)	2 years (2012–2013)
Sampling interval	5 min	1 h
Total time series length	315,648 time steps	17,544 time steps
Number of meteorological variables	5	5
Number of temporal samples	315,552	17,448

**Table 3:** Overall forecasting performance comparison on wind power datasets. Results are presented as mean ± standard deviation over five independent runs. **Bold** indicates optimal performance. The * symbol indicates statistical significance at p<0.05 compared to the best-performing baseline using a paired *t*-test.

Method	MAE	RMSE	MAPE (%)	PICP	MPIW	CWC	CRPS
Persistence Model	0.142±0.008	0.198±0.010	18.73±0.85	0.61±0.04	0.284±0.012	0.312±0.014	0.115±0.005
ARIMA	0.128±0.006	0.183±0.008	16.45±0.72	0.64±0.03	0.271±0.010	0.298±0.011	0.102±0.004
SVR	0.119±0.005	0.172±0.007	15.02±0.65	0.66±0.03	0.265±0.008	0.291±0.010	0.095±0.004
Random Forest	0.113±0.005	0.165±0.006	14.36±0.58	0.69±0.02	0.258±0.007	0.283±0.009	0.089±0.003
LSTM	0.105±0.004	0.154±0.005	13.21±0.52	0.72±0.02	0.246±0.006	0.271±0.008	0.082±0.003
GRU	0.103±0.004	0.151±0.005	12.98±0.48	0.73±0.02	0.241±0.006	0.268±0.007	0.079±0.003
Temporal CNN	0.101±0.003	0.148±0.004	12.67±0.45	0.74±0.02	0.239±0.005	0.265±0.006	0.077±0.002
Deterministic Transformer	0.097±0.003	0.143±0.004	12.11±0.41	0.75±0.02	0.236±0.004	0.263±0.005	0.074±0.002
Informer	0.095±0.003	0.141±0.003	11.85±0.38	0.82±0.02	0.230±0.004	0.255±0.005	0.071±0.002
PatchTST	0.092±0.002	0.136±0.003	11.20±0.35	0.86±0.01	0.226±0.003	0.248±0.004	0.068±0.002
Proposed Method	0.089±0.002 *	0.132±0.002 *	10.84±0.28 *	0.91±0.01 *	0.221±0.002 *	0.241±0.003 *	0.062±0.001 *

**Table 4:** Comparison with probabilistic forecasting methods. Results are presented as mean ± standard deviation over five independent runs. **Bold** indicates optimal performance. The * symbol indicates statistical significance at p<0.05 compared to the best-performing baseline using a paired *t*-test.

Method	PICP	MPIW	CWC	RMSE
Quantile Regression	0.87±0.02	0.248±0.007	0.276±0.009	0.156±0.006
Bayesian Neural Network	0.89±0.02	0.255±0.008	0.281±0.010	0.152±0.005
MC Dropout	0.88±0.02	0.243±0.006	0.272±0.008	0.149±0.005
Interval LSTM	0.90±0.01	0.238±0.005	0.266±0.007	0.147±0.004
Probabilistic Transformer	0.92±0.01	0.234±0.005	0.259±0.006	0.145±0.004
Probabilistic Informer	0.90±0.01	0.230±0.004	0.255±0.006	0.141±0.003
Probabilistic PatchTST	0.91±0.01	0.226±0.004	0.248±0.005	0.136±0.003
Proposed Method	0.91±0.01 *	0.221±0.002 *	0.241±0.003 *	0.132±0.002 *

**Table 5:** Ablation study on key components of the proposed framework. Results are presented as mean ± standard deviation over five independent runs. **Bold** indicates optimal performance.

Configuration	MAE	RMSE	MAPE (%)	PICP	MPIW	CWC
Full model	**0.089** ± **0.002**	**0.132** ± **0.002**	**10.84** ± **0.28**	**0.91** ± **0.01**	**0.221** ± **0.002**	**0.241** ± **0.003**
w/o probability-driven attention	0.097±0.003	0.145±0.004	12.03±0.35	0.83±0.02	0.227±0.004	0.269±0.006
w/o multi-scale period modeling	0.094±0.003	0.139±0.003	11.54±0.32	0.86±0.02	0.231±0.004	0.258±0.005
w/o quantile prediction head (point only)	0.091±0.002	0.134±0.003	11.02±0.30	–	–	–
w/o time-slice GCN	0.093±0.003	0.138±0.003	11.47±0.31	0.87±0.02	0.229±0.003	0.256±0.005
w/o channel-correlation GCN	0.095±0.003	0.141±0.003	11.81±0.33	0.85±0.02	0.233±0.004	0.262±0.005
w/o dynamic $u_{j}$ computation	0.095±0.003	0.142±0.004	11.85±0.34	0.84±0.02	0.228±0.004	0.265±0.006
w/o learnable γ	0.092±0.002	0.136±0.003	11.18±0.31	0.88±0.01	0.224±0.003	0.250±0.004

**Table 6:** Fine-grained ablation study on the Quantile Prediction and Interval Modeling module. Results are presented as mean ± standard deviation over five independent runs. **Bold** indicates optimal performance. The * symbol indicates statistical significance at p<0.05 compared to the ablated baselines using a paired *t*-test.

Configuration	MAE	RMSE	MAPE (%)	PICP	MPIW	CWC
Full Module	**0.089** ± **0.002** *	**0.132** ± **0.002** *	**10.84** ± **0.28** *	**0.91** ± **0.01** *	**0.221** ± **0.002** *	**0.241** ± **0.003** *
w/o Temporal GCN	0.093±0.003	0.138±0.003	11.47±0.31	0.87±0.02	0.229±0.003	0.256±0.005
w/o Channel GCN	0.095±0.003	0.141±0.003	11.81±0.33	0.85±0.02	0.233±0.004	0.262±0.005
MLP Only (w/o Both GCNs)	0.098±0.004	0.146±0.004	12.15±0.36	0.82±0.02	0.238±0.005	0.274±0.007
w/o Monotonic Constraint	0.091±0.002	0.135±0.003	11.08±0.30	0.88±0.02	0.235±0.004	0.252±0.004

**Table 7:** Ablation study on attention formulation and data augmentation strategies (Results reported as mean ± standard deviation over 5 independent runs). **Bold** indicates optimal performance.

Model Configuration	RMSE	MAE	PICP	MPIW
Proposed (Additive Formulation)	**0.132 ± 0.002**	**0.089 ± 0.001**	**0.910 ± 0.005**	**0.221 ± 0.004**
Multiplicative Gating	0.136 ± 0.003	0.092 ± 0.002	0.895 ± 0.008	0.230 ± 0.006
Temperature Scaling	0.134 ± 0.002	0.090 ± 0.001	0.880 ± 0.007	0.215 ± 0.005
w/o Augmentation	0.141 ± 0.004	0.096 ± 0.003	0.875 ± 0.010	0.245 ± 0.008

## Data Availability

The data presented in this study are available on request from the corresponding author.
